# Supplementary material for: Organo-Vermiculites Modified by Aza-Containing Gemini Surfactants: Efficient Uptake of 2-Naphthol and Bromophenol Blue
Source: Nanomaterials (Basel). 2022 Oct 17;12(20):3636. doi: 10.3390/nano12203636 (PMC9609671; doi:10.3390/nano12203636)
Supplement: Supplementary file 1 [file nanomaterials-12-03636-s001.zip › nanomaterials-1951625-supplementary.pdf]

nanomaterials MDPI

Supplementary Materials

Organo-Vermiculites Modified by Aza-Containing Gemini Surfactants: Efficient Uptake of 2-Naphthol and Bromophenol Blue

Jianchao Gong [1], Tingting Wang [1,2], Wei Zhang [3], Lin Han [1,2], Mingxiao Gao [4], Tianen Chen [1], Tao Shen [1,*] and Yaxiong Ji [1,*]

[1] Hoffmann Institute of Advanced Materials, Shenzhen Polytechnic, 7098 Liuxian Boulevard, Nanshan District, Shenzhen 518055, China
[2] Department of Physics, Innovation Laboratory of Materials for Energy and Environment Technologies, College of Science, Tibet University, Lhasa 850000, China
[3] PetroChina Petrochemical Research Institute, Beijing 102206, China; zhangwei020@petrochina.com.cn
[4] Anshan No. 1 Middle School, Anshan 114051, China
* Correspondence: shentao19931026@163.com (T.S.); kobejyx@126.com (Y.J.); Tel.: +(86)-1581-8696-045 (Y.J.)

1. Characterization

The FT-IR spectroscopy (FT-IR, Nicolet IS10 FT-IR spectrometer, USA), X-ray diffractometer (XRD, BRUCKER D8 ADVANCE, Germany), thermogravimetric analysis (TG-DTG, NETZSCH STA 449 F5/F3 Jupiter, Germany), EA (Vario EL cube, Germany) and surface area analysis (BET, ASAP 2460, USA) were conducted to for structural characterization. Zeta potential of organo-Vt were recorded by immersing 0.03 g of organo-Vt in water at various pH values (Zetasizer Nano ZS90, Netherlands).

The structural characters of organo-Vts were characterized by: Fourier transformed infrared spectroscopy (FT-IR, Nicolet Magna 560 E.S.P FT-IR (USA) spectrometer in the range of 4000-400 cm^{-1} at resolution of 4 cm^{-1}), X-ray diffractometer (XRD, in the 2θ range from 1° to 10° at the scanning rate of 1° min^{-1}), thermogravimetric analysis (TG-DTG, METLER TOLEDO, from 30 to 800 °C at 10 °C min^{-1}, nitrogen atmosphere), elemental analysis (EA, Vario MACRO cube, Germany) and scanning electron microscopy-energy dispersive X-ray spectroscopy (SEM-EDS, Quanta 200F field emission scanning electron microscope (USA), samples are dispersed onto the conducting resin). Zeta potentials of the samples were measured on a Zetasizer Nano ZS. All samples were all dried at 60 °C overnight before characterization.

2. Adsorption experiment

Batch adsorption experiments were adopted for evaluating the adsorption performance of organo-Vts. Analysis about the influence factor (modifier dosage, adsorption time, initial CR concentration, temperature and solution pH) and adsorption mechanism (adsorption kinetics, isotherms and thermodynamics) was conducted by mixing 0.03 g of organo-Vts and 30 mL of CR solution under varying conditions, respectively. The concentration CR is measured by UV-vis. The adsorption amounts (q_e, mg g^{-1}) were obtained by the following equation (Equation S1):

$$q_e = \frac{C_0 - C_e}{m} V \qquad \text{(S1)}$$

where q_e is the adsorption amount onto adsorbent (mg/g), C_0 and C_e are the initial and equilibrium dye concentrations (mg/L), respectively, m represents the mass of adsorbent (g) and V stands for the volume of solution (mL).

3. The adsorption kinetics, isotherms and thermodynamics

The equations of pseudo-first- (S2), pseudo-second order (S3) and intra particle diffusion (S4) models, the Langumir (S5), Freundlich (S6) and Redlich-Peterson (S7), as well as thermodynamic parameters (S8 and S9) are expressed as follows:

$$\log(q_e - q_t) = \log q_e - \frac{k_1}{2.303}t \tag{S2}$$

$$\frac{t}{q_t} = \frac{1}{k_2 q_e^2} + \frac{1}{q_e}t \tag{S3}$$

$$q_t = k_i t^{1/2} + C \tag{S4}$$

where k_1 (min^{-1}) and k_2 (g (mg min)$^{-1}$) were the pesudo-first-order and pesudo-second-order rate constants. q_e (mg g^{-1}) and q_t (mg g^{-1}) were the adsorption capacities at equilibrium and at time t (min),which represents contact time. All these unknown parameters can be determined from plots of log (q_e - q_t) against t and t/q_t against t. k_{id} (mg g^{-1} min$^{-1/2}$) is the rate constant of the intra-particle
diffusion kinetic model, the values of C and k_{id} can be determined from the intercept and slope of the linear plotted of q_t against $t^{1/2}$, respectively.

The Langmuir, Freundlich and Redlich-Peterson isotherms were expressed as follows:

$$q_e = \frac{Q_m K_L C_e}{1 + K_L C_e} \tag{S5}$$

$$q_e = K_f C_e^{1/n} \tag{S6}$$

$$q_e = \frac{A C_e}{1 + B C_e^g} \tag{S7}$$

where q_e (mg/g) is the adsorption capacity onto per unit mass of adsorbent at equilibrium, C_e is the solute equilibrium concentration (mg/L), q_m (mg/g) is maximum adsorbed amount in the theoretical. k_L (L/mg), k_f (mg/g) and n represent the constants of Langmuir and Freundlich, respectively. A (L/g) and B ((L/mg)^g) are the Redlich–Peterson model constant. g fluctuated between 0 and 1 with two limiting behaviors: Langmuir form for g = 1 and Henry's law form for g = 0.

Thermodynamic parameters could be calculated using the following equations:

$$\ln K_L = \frac{\Delta S^\circ}{R} - \frac{\Delta H^\circ}{RT} \tag{S8}$$

$$\Delta G^\circ = -RT \ln K_L \tag{S9}$$

where K_L is the Langmuir constant, q_e and C_e have the same definitions with above equations. R is the universal gas constant (8.3145 J/(mol K)) and T represents the absolute temperature in Kelvin. The values of ΔH° and ΔS° can be extrapolated from intercept and slop of ln K_L versus 1/T.

4. Theoretical Calculation

Quantum chemical calculations were conducted on Material Studio 8.0. The optimized structures and molecular frontier orbitals were calculated based on density functional theory (DFT). Dmol3 module was adopted for calculation, where the function

GGA&BLYP (generalized gradient approximation & Becke-Lee-Yang-Parr) was used in all processes. The Adsorption Locator module was applied to simulate the optimized adsorption configuration, and the forcefield was set to universal.

$$\Delta N=(\chi_1-\chi_2)/2(\zeta_1+\zeta_2) \quad (S10)$$

$$\zeta=(I-E_A)/2 \quad (S11)$$

$$\chi=(I+E_A)/2 \quad (S12)$$

$$I=-E_{HOMO} \quad (S13)$$

$$E_A=-E_{LUMO} \quad (S14)$$

Table S1. Elemental analysis and ^{1}H NMR data of 5N, 7N and 8N.

Samples	Elemental analysis (wt%)						^{1}H NMR analysis
	C		H		N		
	Cal.	Test	Cal.	Test	Cal.	Test	
5N	58.02	57.33	11.48	9.65	6.26	6.17	δ 0.88 (6H), 1.26 (32H), 1.29 (4H), 1.71 (4H), 2.18 (3H), 2.80 (4H), 3.22 (4H), 3.30 (12H), 3.34 (4H)
7N	59.09	54.59	11.58	10.29	6.01	5.19	δ 0.88 (6H), 1.26 (32H), 1.29 (4H), 1.71 (4H), 1.83 (4H), 2.18 (3H), 2.46 (4H), 3.22 (8H), 3.30 (12H)
8N	59.99	56.19	11.68	10.24	8.01	7.07	δ 0.88 (6H), 1.26 (32H), 1.29 (4H), 1.71 (4H), 2.18 (6H), 2.37 (4H), 2.80 (4H), 3.22 (8H), 3.30 (12H), 3.34 (4H)

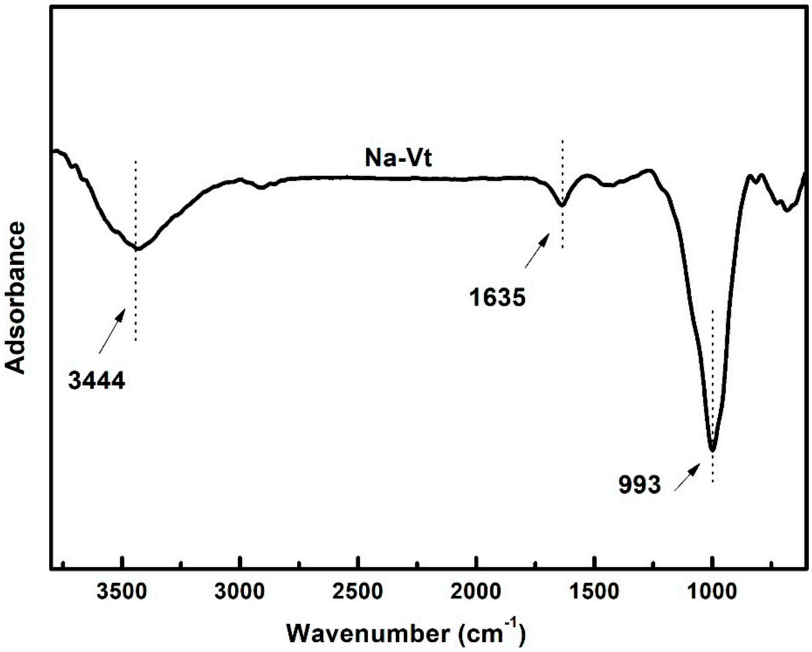

Figure S1. The FT-IR spectrum of Na-Vt.

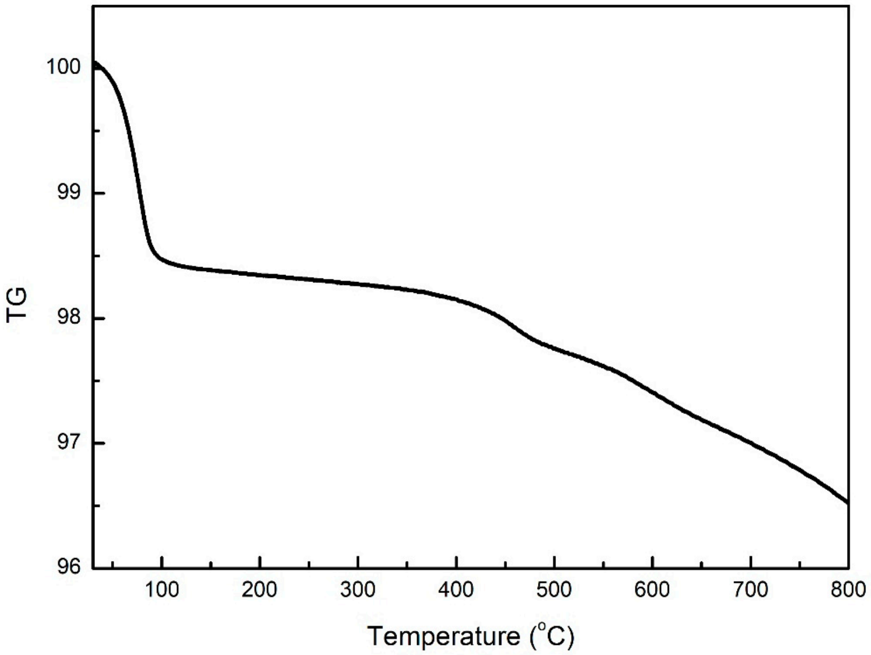

Figure S2. The TG curve of Na-Vt.

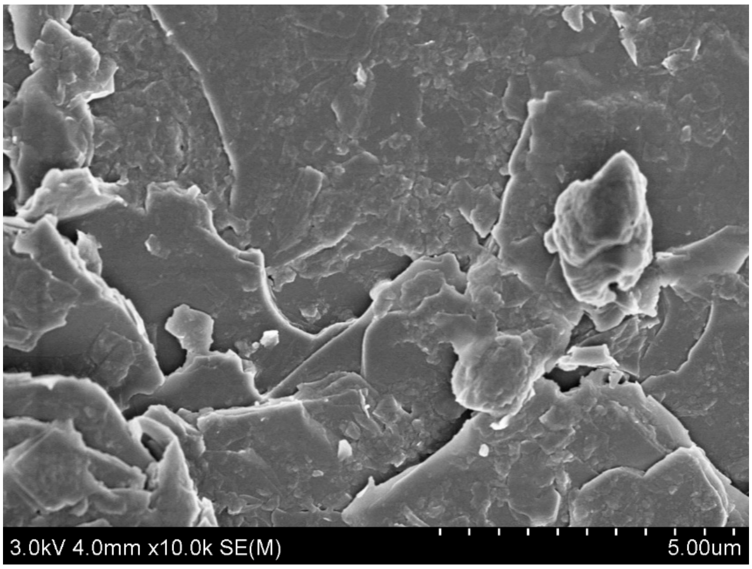

Figure S3. The SEM image of Na-Vt.

Table S2. Zeta potential values of 5N-Vt, 7N-Vt and 8N-Vt.

Samples	pH				
	3	5	7	9	11
5N-Vt	−16.8	−20.2	−20.7	−20.0	−23.4
7N-Vt	−6.47	−19.6	−19.9	−19.6	−23.9
8N-Vt	−5.71	−16.4	−16.0	−15.9	−26.2
